# Using Machine Learning and Finite Element Analysis to Extract Traction-Separation Relations at Bonding Wire Interfaces of Insulated Gate Bipolar Transistor Modules

**DOI:** 10.3390/ma17051002

**Published:** 2024-02-22

**Authors:** Shengjun Zhao, Tong An, Qi Wang, Fei Qin

**Affiliations:** 1 Institute of Electronics Packaging Technology and Reliability, School of Mathematics, Statistics and Mechanics, Beijing University of Technology, Beijing 100124, China; sjzhao@emails.bjut.edu.cn (S.Z.); wangqi169@emails.bjut.edu.cn (Q.W.); qfei@bjut.edu.cn (F.Q.); 2 Beijing Key Laboratory of Advanced Manufacturing Technology, Beijing University of Technology, Beijing 100124, China

**Keywords:** IGBT, bonded interface mechanical property, traction-separation relations, cohesive zone model, machine learning

## Abstract

For insulated gate bipolar transistor (IGBT) modules using wire bonding as the interconnection method, the main failure mechanism is cracking of the bonded interface. Studying the mechanical properties of the bonded interface is crucial for assessing the reliability of IGBT modules. In this paper, first, shear tests are conducted on the bonded interface to test the bonded interface’s strength. Then, finite element–cohesive zone modeling (FE-CZM) is established to describe the mechanical behavior of the bonded interface. A novel machine learning (ML) architecture integrating a convolutional neural network (CNN) and a long short-term memory (LSTM) network is used to identify the shape and parameters of the traction separation law (TSL) of the FE-CZM model accurately and efficiently. The CNN-LSTM architecture not only has excellent feature extraction and sequence-data-processing abilities but can also effectively address the long-term dependency problem. A total of 1800 sets of datasets are obtained based on numerical computations, and the CNN-LSTM architecture is trained with load–displacement (*F*–*δ*) curves as input parameters and TSL shapes and parameters as output parameters. The results show that the error rate of the model for TSL shape prediction is only 0.186%. The performance metric’s mean absolute percentage error (MAPE) is less than 3.5044% for all the predictions of the TSL parameters. Compared with separate CNN and LSTM architectures, the proposed CNN-LSTM-architecture approach exhibits obvious advantages in recognizing TSL shapes and parameters. A combination of the FE-CZM and ML methods in this paper provides a promising and effective solution for identifying the mechanical parameters of the bonded interfaces of IGBT modules.

## 1. Introduction

With their fast switching speed, low driving power consumption, and simple driving circuit, IGBT modules are key devices in power electronic systems [1]. Wire bonding is the main interconnection method of IGBT modules and is used to connect chips and external circuits [2]. In practical applications, the bonded interface is subjected to large thermal stresses due to the difference between the coefficients of thermal expansion (CTEs) of the Al bonding wires and the Si chip. As the service time increases, cracks develop at the bonded interface and gradually expand, degrading the mechanical properties of the bonded interface [3]. The degradation of the mechanical properties of the bonded interface seriously affects the conductive and heat transfer characteristics of the IGBT module and ultimately leads to the IGBT module failing. Studies have shown that cracking of the bonded interface is one of the main failure mechanisms of IGBT modules [4]. Therefore, studying the mechanical properties of bonded interfaces is crucial for assessing the reliability of IGBT modules.

The mechanical properties of interfaces have been studied primarily through experimental measurements or numerical simulations. The fracture toughness of pure type I/II interfaces is typically experimentally measured via uniaxial tensile tests, shear tests, and end-notched flexural (ENF) tests [5]. The mixed-mode bending (MMB) test is used to measure the interfacial fracture characteristics of the mixed mode [6]. However, as the problem becomes more complex, especially under certain practical working conditions, measuring the mechanical properties of the interface through experiments becomes very difficult and expensive [7]. Therefore, several researchers have adopted numerical simulation methods to characterize the mechanical properties of interfaces. The cohesive zone modeling (CZM) method proposed by Dugdale [8] and Barenblatt [9] is the most popular method and has been widely used to address interface problems involving different structures [10,11,12,13].

In the CZM method, the mechanical behavior of the interface is described by the traction-separation law (TSL). Different TSLs, including the bilinear law [14]; the trapezoidal law [15]; the polynomial law [16]; the exponential potential-based law [17]; and the Park, Paulino, and Roesler (PPR) law [18], have different shapes and corresponding parameters. Xu [19] noted that the effect of the TSL shape generally cannot be ignored, especially for type II fractures. The literature [20,21,22] has summarized the general rules for the influence of the TSL shape. Depending on the material and interface to be simulated, the appropriate TSL (both shape and parameters) must be chosen to make reasonable predictions. Once the TSL shapes and parameters of the bonding interface of IGBT modules are accurately predicted, the health status can be made clear, and further guidance for service reliability can be provided.

Several researchers have used the CZM method to study the bonded interfaces of IGBT modules. Halouani [23] analyzed the degradation behavior of bonding wires using the bilinear law. Luo [24] combined the bilinear law with the multiscale approach to study the crack extension of bonding wires. Shqair [25] established the relationship between the parameters in the bilinear law and the microstructural properties and predicted the crack paths of the bonded interfaces. However, the current studies on bonded interfaces for IGBT modules empirically select TSLs without analyzing the effect of the shape, which may lead to errors.

Methods for determining the TSL parameters can be divided into direct and indirect methods [26,27]. Direct methods use experimental tests, such as double cantilever beam (DCB) tests [26], to determine the TSL parameters. This method is not applicable here, because fabricating specimens with double cantilever beam structures at the bonded interface of IGBT modules is difficult. The indirect method is an inverse analysis method [19,28,29] based on finite element (FE) simulation, where the appropriate TSL is determined by comparing the numerical solution with the experimental measurements. Maier [30,31], using an inverse analysis strategy, estimated residual stress and identified the elastic–plastic material parameters. However, this method requires continuous trial and error, is computationally inefficient, and is dependent on the initial parameter estimation. In addition, extensive numerical simulations are required for each experimental result. This has motivated the search for more generalized methods for determining TSL parameters.

Machine learning (ML) technology has been developing rapidly in recent years. Its advantages in terms of its block learning speed, its simple model structure, and its ability to manage larger data volumes and nonlinear mapping make it a promising method for identifying TSL parameters. This method may also be described as an inverse analysis strategy. Su [32] used an artificial neural network (ANN) model based on exponential law to predict the TSL parameters of the interface between fiber-reinforced polymers. Hou [33] used a generalized regression neural network (GRNN) model to predict the TSL parameters of a mixed-mode thermal barrier coating system based on the bilinear law. Similarly, the Gaussian process regression (GPR) algorithm [34], the random forest regression (RFR) model [35], and dynamic convolutional neural network (DCNN) [36] have been used to predict various TSL parameters. The ML models in the above studies achieve strong TSL parameter-prediction results. However, in most of the currently established ML models, only a single shape of the TSL is considered, and the effect of the TSL shape is not considered. In addition, the load–displacement (*F*–*δ*) curve itself has a strong time dependence, and recent research fails to consider the time-series characteristics of the *F*–*δ* curve as an input parameter. As a result, the established ML models have poor application potentials. Long short-term memory (LSTM) machine learning algorithms, which can effectively handle sequential data and can learn the long-term dependence of the data [37], have been used to consider time-series features of *F*–*δ* curves. However, Long’s study [38] revealed that when LSTM networks were used alone for prediction, the results were only acceptable and did not yield the expected excellent results.

To solve the above problems, in this paper, a CNN-LSTM architecture combining a convolutional neural network (CNN) and LSTM [39] is used for the first time to estimate TSLs at the bonded interface of IGBT modules. Utilizing the powerful feature-extraction capability of CNNs, the features of the original data are first extracted and subsequently used as inputs to the LSTM for prediction. The proposed method can fully utilize the time-series features of the recorded data and can extract data features and long-term memory curves.

**Figure 1:**
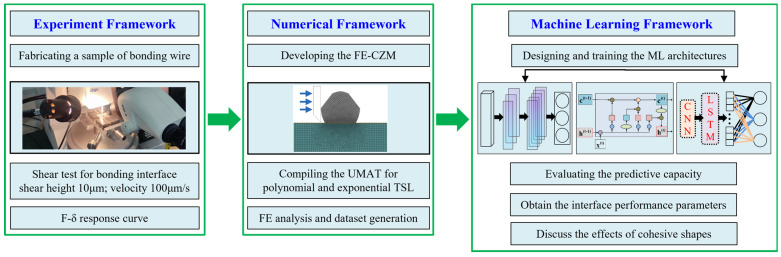
Schematic of the research framework used in this study.

To accurately characterize the mechanical properties of the bonding wire interfaces of IGBT modules, in this work, an experimental study is first conducted on the bonded interfaces to obtain the *F*–*δ* curve response of the bonded interfaces. Then, based on the tests, the FE-CZM model of the bonded region is established. Three different shapes of TSLs are used, i.e., the bilinear law, exponential law, and polynomial law. Based on the numerical simulation, 1800 datasets are obtained to train the CNN-LSTM architecture with *F*–*δ* curves as the input parameters and TSL shapes and TSL parameters as the output parameters. The prediction performance of the constructed CNN-LSTM model is evaluated using the coefficient of determination (*R*^2^), the root mean square error (RMSE), the mean absolute percentage error (MAPE), and the error-rate metric. The results of the proposed model are compared with those of the CNN and LSTM architecture. Finally, based on the experimentally obtained *F*–*δ*-curve responses, the TSL shapes and TSL parameters characterizing the mechanical properties of the bonded interface of the IGBT modules are discussed, and the effectiveness of the methodology is verified by comparing the experimental data and the predicted results. The research schematic used in this study is depicted in Figure 1.

## 2. Experimental Procedure

To evaluate the mechanical properties of the bonded interface of the IGBT modules, shear tests were conducted on the bonding wires. The *F*–*δ*-curve responses obtained from the tests were used to compare the predicted results and those of the numerical simulations to validate the prediction ability of the CNN-LSTM architecture.

**Figure 2:**
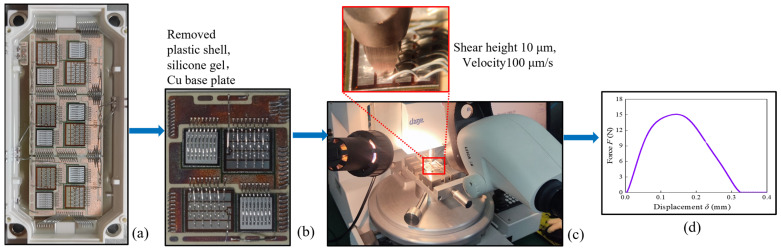
Shear test process: (**a**) the IGBT module; (**b**) the shear test sample; (**c**) testing platform; (**d**) *F*–*δ*-curve response.

In the shear tests, the device utilized was a 1200 V/450 A IGBT module. The module consists of a plastic case, silicone gel, a Cu backplate, a solder layer, direct bonding copper (DBC) ceramic base plates, bonding wires, a freewheel diode (FWD) chip, and an IGBT chip, as shown in Figure 2a. Before the shear test, the plastic shell, silicone gel, and Cu base plates were removed for easy operation. The samples for the shear test were obtained as shown in Figure 2b.

The test equipment used was a DAGE-4000 tensile/shear tester (Nordson, Westlake, OH, USA). The shear height was 10 μm, and the shear speed was 100 μm/s, according to the DVS-2811 standard [40]. The fabricated specimen was placed on the tester, as shown in Figure 2c. During the test, the applied load and the displacement of the shear chisel were recorded in real time, as shown in Figure 2d.

## 3. Cohesive Zone Modeling

**Figure 3:**
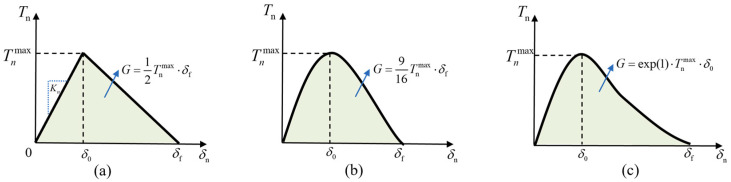
The constitutive relationship of the TSL: (**a**) bilinear; (**b**) polynomial; (**c**) exponential.

To examine the influence of the TSL shape on the mechanical properties of the bonded interface of IGBT modules, three commonly used TSLs [41], namely, the bilinear law, the exponential law, and the polynomial law, are selected in this paper, as illustrated in Figure 3. The bilinear law is available in ABAQUS 2021. The exponential and polynomial laws are implemented based on the user-defined subroutine UMAT. In addition, the FE-CZM model of the bonded region is established, based on which the shear test is simulated to provide a sufficient dataset for training the ML model.

### 3.1. Cohesive Law Description

#### 3.1.1. Bilinear Law

The bilinear law [14] has a triangular shape and assumes an initial linear elastic behavior followed by a linear damage-softening response, as shown in Figure 3a. The relationship between the separating displacement δ and the corresponding traction T on the interface can be expressed as follows.

(1) $$T=\{\begin{array}{ll} \delta \cdot \frac{T^{\max}}{\delta_{0}} & 0\leq \delta \leq \delta_{0}\\ T^{\max}\cdot \frac{\delta_{f}-\delta }{\delta_{f}-\delta_{0}} & \delta_{0}\leq \delta \leq \delta_{f} \end{array}$$

(2) $$T^{\max}=K\cdot \delta_{0}$$

(3) $$G=\frac{1}{2}T^{\max}\cdot \delta_{f}$$

where *δ*_0_ represents the separating displacement corresponding to the maximum traction *T*^max^, *K* represents the stiffness, *δ*_f_ represents the separating displacement at complete failure, and *G* represents the critical fracture energy.

#### 3.1.2. Polynomial Law

The polynomial law [16] differs from the bilinear law in that it is a continuously differentiable function that converges better during numerical simulations. The normal and tangential constitutive relationships are written as follows.

(4) $${Τ}_{n}=\left\{ \begin{array}{ll} \frac{27}{4}T_{n}^{\max}\{\left(\frac{\delta_{n}}{\delta_{f}}\right)\left[1-2\left(\frac{\delta_{n}}{\delta_{f}}\right)+{\left(\frac{\delta_{n}}{\delta_{f}}\right)}^{2}\right]+\alpha {\left(\frac{\delta_{t}}{\delta_{f}}\right)}^{2}[(\frac{\delta_{n}}{\delta_{f}})-1]\} & \delta_{n}\leq \delta_{f}\\ 0 & \delta_{n}>\delta_{f} \end{array}\right.$$

(5) $${Τ}_{t}=\left\{ \begin{array}{ll} \frac{27}{4}T_{n}^{\max}\{\alpha \left(\frac{\delta_{t}}{\delta_{f}}\right)\left[1-2\left(\frac{\delta_{n}}{\delta_{f}}\right)+{\left(\frac{\delta_{n}}{\delta_{f}}\right)}^{2}\right]\} & \delta_{t}\leq \delta_{f}\\ 0 & \delta_{t}>\delta_{f} \end{array}\right.$$

(6) $$T_{n}^{\max}=\frac{16}{9}\frac{G_{n}}{\delta_{f}}$$

(7) $$T_{t}^{\max}=\frac{16}{9}\frac{G_{t}}{\delta_{f}}$$

where *δ*_n_ and *δ*_t_ are the normal and tangential separating displacements, respectively; *T*_n_ and *T*_t_ are the normal and tangential traction components, respectively; $T_{n}^{\max}$ and $T_{t}^{\max}$ are the maximum normal and tangential tractions, respectively; and *G*_n_ and *G*_t_ are the critical normal and tangential fracture energies, respectively. The polynomial law has two independent parameters.

#### 3.1.3. Exponential Law

The exponential law, originally proposed by Needleman [17] and later modified by van den Bosch [42], is a continuously differentiable function. The normal and tangential constitutive relationships are as follows.

(8) $$T_{n}=\frac{G_{n}}{\delta_{0}}\left(\frac{\delta_{n}}{\delta_{0}}\right)\exp\left(-\frac{\delta_{n}}{\delta_{0}}\right)\exp\left(-{\left(\frac{\delta_{t}}{\delta_{0}}\right)}^{2}\right)$$

(9) $$T_{t}=2\frac{G_{t}}{\delta_{0}}\left(\frac{\delta_{t}}{\delta_{0}}\right)\left\{1+\frac{\delta_{n}}{\delta_{0}}\right\}\exp\left(-\frac{\delta_{n}}{\delta_{0}}\right)\exp\left(-{\left(\frac{\delta_{t}}{\delta_{0}}\right)}^{2}\right)$$

(10) $$T_{n}^{\max}=\frac{G_{n}}{\exp(1)\cdot \delta_{0}}$$

(11) $$T_{t}^{\max}=\frac{\sqrt{2}G_{t}}{\delta_{0}\sqrt{\exp(1)}}$$

### 3.2. FE-CZM for Bonded Interfaces

**Figure 4:**
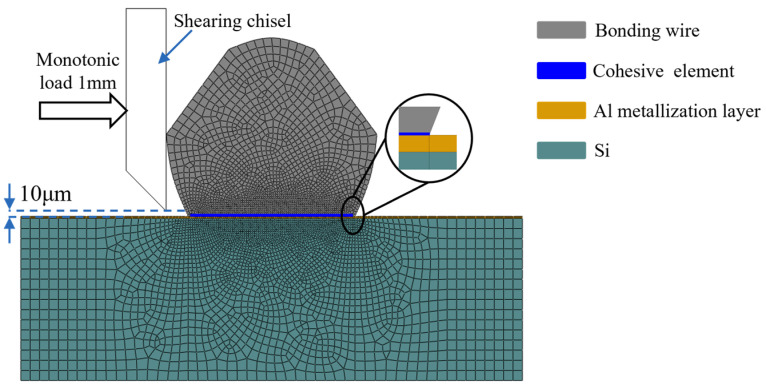
Schematic of the FE mesh model.

A 2D FE model is established to simulate the bonding wire shear test, as shown in Figure 4. This model consists of a shear chisel, a bonding wire, an Al metallization layer, and a chip. The dimensions of the Al metallization layer and the chip are 320 μm × 4 μm and 320 μm × 150 μm, respectively. The dimensions of the bonding wire are the same as those in practice.

The shear chisel is considered an analytically rigid body. Zero-thickness cohesive elements, of type COH2D4, which are 4-node two-dimensional cohesive elements, are inserted at the interface between the Al metallization layer and the bonding wire. Four-node bilinear plane strain elements, of type CPE4 in ABAQUS, are applied for the remainder of the model. The FE-CZM model contains a total of 8304 elements.

All the materials are considered isotropic, and the material properties of Al and Si are shown in Table 1 [24,25]. The boundary conditions and load settings are kept consistent with those of the shear test, with a full constraint applied to the bottom of the chip, and a horizontal displacement load (*δ* = 100 μm) is applied to the shear chisel. To increase the convergence speed and reduce the computational time of this simulation, we assume that the bonding wires can move only horizontally.

Notably, the ABAQUS 2021 software provides only a bilinear cohesive model. The constitutive relationships for the exponential and polynomial laws are not given and must be implemented using a user-defined subroutine, UMAT. Therefore, based on the equations given in Section 3.1, the UMAT subroutine for the exponential and polynomial laws is established. In the UMAT subroutine, the nodal force vector is computed and outputted to the main routine of ABAQUS, and the Jacobi matrix is obtained. After the calculation is complete, the required results can be outputted using the state variables defined in advance. Notably, the accuracy of the Jacobi matrix in UMAT affects the convergence speed of the calculation of the ABAQUS main program but does not significantly impact the calculation results.

## 4. Machine Learning Framework

ML has been widely used in different fields, and many different types of ML network architectures exist. The most significant advantage of ML models is that they can make predictions about future data, provided that the model performs well on a testing dataset. However, the ability of a machine learning algorithm to perform well depends on whether the algorithm matches the problem itself. Therefore, based on the problem to be solved in this paper, we choose an ML architecture that combines a CNN and LSTM to fully utilize the advantages of these two algorithms. In this section, the CNN, LSTM, and CNN-LSTM algorithms are given, and the network architecture is built. In addition, the implementation process of the ML architecture, including data collection, the ML architecture, and performance metrics, is presented.

### 4.1. Data Collection

Collecting sufficient data plays a significant role in the ML model training process. The dataset generated by the FE-CZM model, considering different combinations of TSL shapes and parameters, is utilized to train the developed ML architecture. The stiffness, maximum traction, and critical fracture energy are randomly selected between 10~900 N/mm^3^, 1~90 MPa, and 0.01~15 N/mm, respectively. Notably, the randomly chosen parameters follow a normal distribution, which ensures the generalizability of the proposed ML architecture. Through a numerical simulation of the FE-CZM model based on the bilinear law, exponential law, and polynomial law, 1000, 400, and 400 datasets, respectively, were collected.

For ML training, different datasets must be selected when the output is the TSL shape or is the TSL parameter. When the output is the TSL shape, all 1800 sets of data are selected as the dataset, 70% of which form the training dataset and 30% of which form the testing dataset. When the output is a bilinear law parameter, 1000 sets of data from the numerical simulations obtained using the bilinear law are selected as the dataset, 70% of which form the training dataset and 30% of which form the testing dataset. Similarly, for exponential and polynomial law parameters, 400 and 400 sets of data obtained from numerical simulations performed using the exponential and polynomial law, respectively, are selected as the dataset, where 70% of each set is used for training and 30% of each set is used for testing.

The inputs of the ML architecture are the *F*–*δ* response curves obtained from FE-CZM simulations. The *F*–*δ* curves are represented as equally spaced (*δ*/*n*) one-dimensional arrays {*x*_1_, *x*_2_, *x*_3_ … *x_n_*}, where *n* is the dimensionality of the response, i.e., the number of features, which is set as *n* = 200 here. The outputs of the ML architecture are the shapes and parameters of the TSLs. Since the shapes are discrete values and the parameters are continuous values, they are predicted using classification and regression techniques, respectively. To improve the convergence speed and accuracy of the network, all the features are normalized before training is conducted, as follows:

(12) $$x_{i}^{\mathrm{new}}=\frac{x_{i}-\min(x_{i})}{\max(x_{i})-\min(x_{i})}\;i=1,2,3,\cdots ,n$$

where $x_{i}$ and $x_{i}^{\mathrm{new}}$ are the original and scaled features, respectively. $\min(x_{i})$ and $\max(x_{i})$ are the minimum and maximum values of the *i*th feature in the training dataset, respectively.

### 4.2. ML Architecture

In this section, three different ML architectures are created, namely, the CNN, LSTM, and CNN-LSTM architectures. These architectures are described below. Each network architecture is used separately for classification (TSL shape prediction) and regression (TSL parameter prediction) analyses. Compared to the ML architecture for regression, the ML architecture for classification has an additional Softmax layer (after the fully connected layer); the rest of the structures are otherwise identical.

#### 4.2.1. CNN Architecture

**Figure 5:**
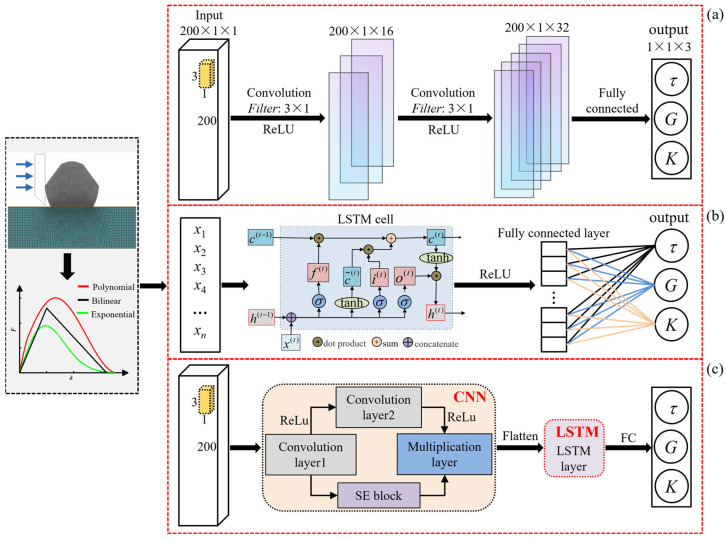
CNN and LSTM architecture: (**a**) CNN; (**b**) LSTM; (**c**) CNN-LSTM.

A CNN generally consists of convolutional, convergent, and fully connected layers with two important properties: local connectivity and weight sharing. The convolutional layer is employed to extract the features of a local region, and the pooling layer is employed to reduce the number of features to avoid overfitting. The CNN is constructed using the convolution operation as follows.

(13) $$y^{\left(l\right)}=\omega^{\left(l\right)}⊗a^{\left(l-1\right)}+b^{\left(l\right)}$$

where *y*^(*l*)^ is the net input of layer *l*; *a*^(*l*−1)^ is the activity value of layer *l* − 1; $\omega^{\left(l\right)}\in {\mathbb{R}}^{K}$ is the convolutional kernel of layer *l*, i.e., the weight vector that can be learned; *K* is the size of the convolutional kernel; and $b^{\left(l\right)}\in {\mathbb{R}}^{K}$ is the learnable bias term of layer *l*. Figure 5a shows the CNN architecture built in this paper, which consists of one fully connected layer and two convolutional layers.

#### 4.2.2. LSTM Architecture

LSTM [35] is a variant of recurrent neural networks that is better able to learn data with long-term dependencies and can achieve optimal performance on challenging sequence-processing tasks. The main arithmetic process of the LSTM network architecture is as follows: first, using the external state *h*^(*t*−1)^ of the previous moment and the input *x*^(*t*)^ of the current moment, the external input gate *i*^(*t*)^, the forget gate *f*^(*t*)^, the output gate *o*^(*t*)^, and the candidate states ${\tilde{c}}^{\left(t\right)}$ are computed, as shown in Equation (14). Notably, the value of “gate” in the LSTM is in the range of (0, 1). Then, the internal state *c*^(*t*)^ is updated by incorporating the memory cell *c*^(*t*−1)^ of the previous moment, as shown in Equation (15). Finally, by incorporating the output gate *o*^(*t*)^, the information of the internal state is transferred to the external state *h*^(*t*)^, as shown in Equation (16). Figure 5b shows the LSTM architecture built in this paper, which consists of one LSTM layer and one fully connected layer.

(14) $$\left[\begin{array}{l} i^{\left(t\right)}\\ f^{\left(t\right)}\\ o^{\left(t\right)}\\ {\tilde{c}}^{\left(t\right)} \end{array}\right]=\left[\begin{array}{c} \sigma \\ \sigma \\ \sigma \\ \tanh \end{array}\right]\left(b+Ux^{\left(t\right)}+Wh^{\left(t-1\right)}\right)$$

(15) $$c^{\left(t\right)}=f^{\left(t\right)}⊙c^{\left(t-1\right)}+i^{\left(t\right)}⊙{\tilde{c}}^{\left(t\right)}$$

(16) $$h^{\left(t\right)}=o^{\left(t\right)}⊙\tanh c^{\left(t\right)}$$

where $b\in {\mathbb{R}}^{4D}$ is the bias, $x_{t}\in {\mathbb{R}}^{4D}$ is the input value at the current moment, and $U,W\in {\mathbb{R}}^{4D\times (M+D)}$ is the weight of the three gates and the candidate state.

#### 4.2.3. CNN-LSTM Architecture

Figure 5c shows the proposed CNN-LSTM architecture. This architecture primarily consists of an input layer, a CNN block, an LSTM block, and an output layer. The CNN block consists of a convolutional network with two paths. One path comprises two convolutional layers, i.e., Convolution 1 and Convolution 2, and the other path comprises a convolutional layer, Convolution 1, and a squeeze-and-excitation (SE) block. The convolutional layer utilizes a 2D convolution with a convolutional kernel size of (3, 1) and a ReLU activation function. The SE block [43] consists of a global average pooling layer, a fully connected layer, a ReLU layer, a fully connected measurement layer, and a sigmoid activation function. The effectiveness of this block has been demonstrated, and optimal performance has been achieved in several tasks [39]. This block is primarily employed to improve the performance and representation of the ML network by adaptively adjusting the weights of each channel. The output of the CNN block is obtained by multiplying the outputs on the two-path SE block and Convolution 2. The data features of the *F*–*δ* curve are extracted by the CNN block and are inputted into the LSTM block as feature vectors. The LSTM block consists of an LSTM layer and a full linkage layer for learning the relationships between *F*–*δ*-curve data features.

The Adam algorithm is used to optimize the network parameters in the ML architecture and to obtain the optimal solution. The learning rate and number of epochs are set to 0.01 and 500, respectively.

### 4.3. Performance Metrics

To evaluate and compare the performances of the architectures used, we employed three error metrics, i.e., the coefficient of determination (*R*^2^), the root mean square error (RMSE), and the mean absolute percentage error (MAPE), to assess the accuracy of the regression analysis results. And we employed the error-rate metric to assess the accuracy of the classification analysis results. These metrics are defined as follows:

(17) $$R^{2}=1-\left[\frac{\sum_{i=1}^{m}{\left(y^{i}-\hat{y^{i}}\right)}^{2}}{\sum_{i=1}^{m}{\left(y^{i}-\bar{y}\right)}^{2}}\right]$$

(18) $$\mathrm{RMSE}=\sqrt{\frac{1}{m}\sum_{i=1}^{m}{\left(y^{i}-\hat{y^{i}}\right)}^{2}}$$

(19) $$\mathrm{MAPE}=\frac{1}{m}\sum_{i=1}^{m}\left|\frac{y^{i}-\overset{⌢}{y^{i}}}{\overset{⌢}{y^{i}}}\right|\times 100\%$$

(20) $$\mathrm{Error}\;\mathrm{rate}=1-100\left(1-\frac{1}{m}\sum_{i=1}^{m}\left|\frac{y^{i}-\overset{⌢}{y^{i}}}{\overset{⌢}{y^{i}}}\right|\right)$$

where $y^{i}$, $\overset{⌢}{y^{i}}$, and $\bar{y}$ are the actual test values, predicted test values, and average actual values, respectively; and *m* is the number of samples in the dataset.

For each metric, *R*^2^ reflects the degree of agreement between the actual data and the fitted function; the closer the *R*^2^ value is to 1, the better the fit and the better the prediction results of the architecture. RMSE and MAPE indicate the deviation of the predicted value from the actual value; the closer these values are to 0, the better the prediction results of the architecture.

## 5. Results and Discussion

### 5.1. ML Results

#### 5.1.1. TSL Shapes

Table 2 provides the TSL shape-prediction abilities of the three ML architectures, CNN, LSTM, and CNN-LSTM. These results show that the CNN-LSTM architecture has the lowest error rate among the three ML architectures on both the training and testing datasets, with an error rate 2−4 times lower than the other two architectures on the testing dataset. The error rates of all three ML architectures on the training dataset are smaller than those on the testing dataset due to overfitting. However, the CNN-LSTM architecture has the smallest difference between the error rates on the training dataset and the testing dataset, indicating that this architecture most effectively handles the overfitting problem. Notably, the overfitting problem is unavoidable in ML, and the training and testing dataset performances must be aligned as much as possible to minimize the risk of overfitting.

**Figure 6:**
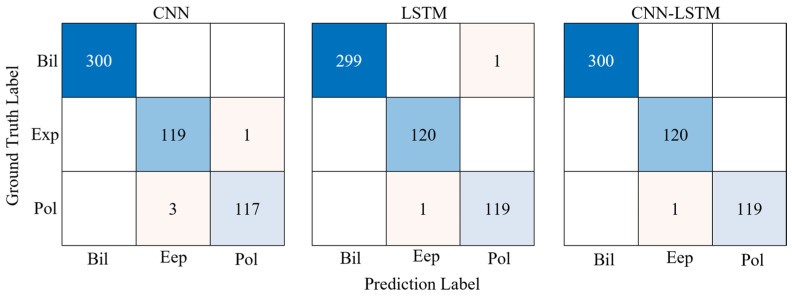
Confusion matrix for the testing dataset.

The confusion matrices of the three ML architectures in the test data are given in Figure 6, where Bil, Exp, and Pol denote the bilinear, exponential, and polynomial laws, respectively. This figure shows that for TSL shape prediction, when the correct prediction result is a bilinear shape, both the CNN and CNN-LSTM architectures can yield the correct prediction result, and the LSTM architecture incorrectly predicts 1 sample as a polynomial shape. When the prediction result is an exponential shape, both the LSTM and CNN-LSTM architectures can yield accurate prediction results, and the CNN architecture incorrectly predicts 1 sample as a polynomial shape. When the correct prediction result is a polynomial shape, both the LSTM and CNN-LSTM architectures incorrectly predict only 1 sample as an exponential shape, while the CNN architecture incorrectly predicts 3 samples as exponential shapes. These results indicate that the CNN-LSTM architecture better predicts TSL shapes than do the individual CNN and LSTM architectures.

#### 5.1.2. TSL Parameters

Table 3 shows the prediction effects of the three ML architectures, CNN, LSTM, and CNN-LSTM, on the corresponding parameters of the three TSL shapes on the testing dataset using the performance metric *R*^2^. The bilinear law corresponds to three parameters, i.e., the stiffness Bil−*K*, the maximum traction Bil−*T*^max^, and the critical fracture energy Bil−*G*; the polynomial law corresponds to two parameters, i.e., the maximum traction Pol−*T*^max^ and the critical fracture energy Pol−*G*; and the exponential law corresponds to two parameters, i.e., the maximum traction Exp−*T*^max^ and the critical fracture energy Exp−*G*. The difference between the actual data and the predicted data is generally considered acceptable when the *R*^2^ value is greater than 0.75. Under this condition, all three ML architectures built in this paper can effectively predict the TSL parameters. As shown by the prediction results of the three ML architectures for each TSL parameter, the CNN-LSTM architecture obtains the largest *R*^2^ value, which indicates that the CNN-LSTM architecture has the best prediction ability among the three ML architectures. Additionally, comparing the predictive abilities of the corresponding parameters of the three TSL shapes reveals that the prediction of the polynomial law parameter is better than that of the bilinear law and exponential law parameters. Among all parameter predictions, the three ML architectures all perform worse for Bil−*G* prediction.

**Figure 7:**
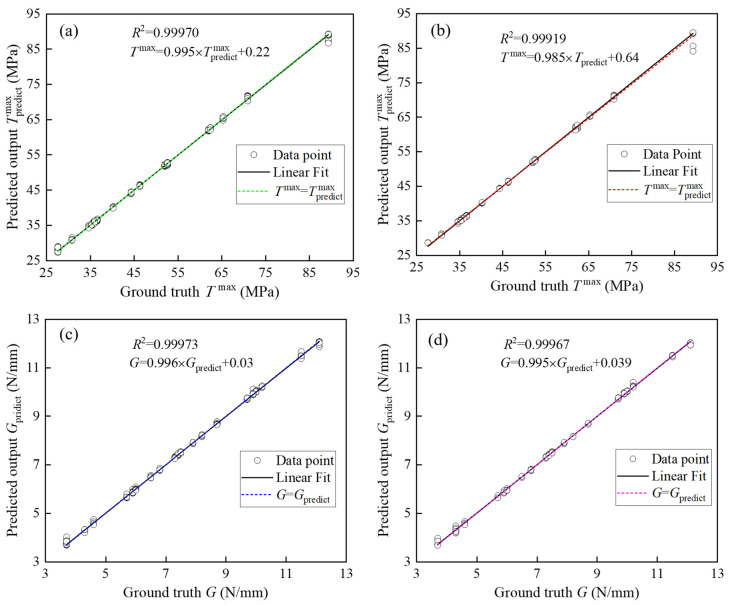
Predicting performance of polynomial law parameters: (**a**) training of Pol−*T*^max^; (**b**) testing of Pol−*T*^max^; (**c**) training of Pol−*G*; (**d**) testing of Pol−*G*.

To evaluate the prediction effect of the CNN-LSTM architecture more intuitively, Figure 7 shows the *R*^2^ values of the prediction effect of the CNN-LSTM architecture using the polynomial law parameters on the training and testing datasets. In the training and test sets, the *R*^2^ values of Pol−*T*^max^ are 0.99970 and 0.99919, and the *R*^2^ values of Pol−*G* are 0.99973 and 0.99967. The predicted values given by the CNN-LSTM architecture thus align well with the true values. The *R*^2^ values of each polynomial law parameter on the training and test sets are almost equal, indicating that the overfitting problem in the CNN-LSTM architecture has been effectively addressed.

**Figure 8:**
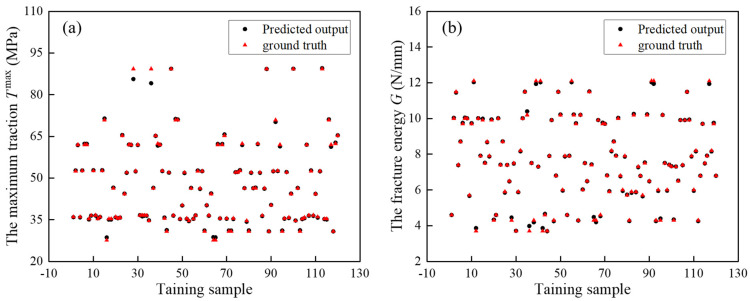
Comparison of the prediction results and ground truth results: (**a**) maximum traction Pol−*T*^max^; (**b**) critical fracture energy Pol−*G*.

To determine the prediction effect of the CNN-LSTM architecture, each predicted and true value on the testing dataset is explicitly given in Figure 8. Most of the predicted values overlap with the true values, indicating that the developed LSTM-CNN model can accurately recognize the parameters of the polynomial law.

**Figure 9:**
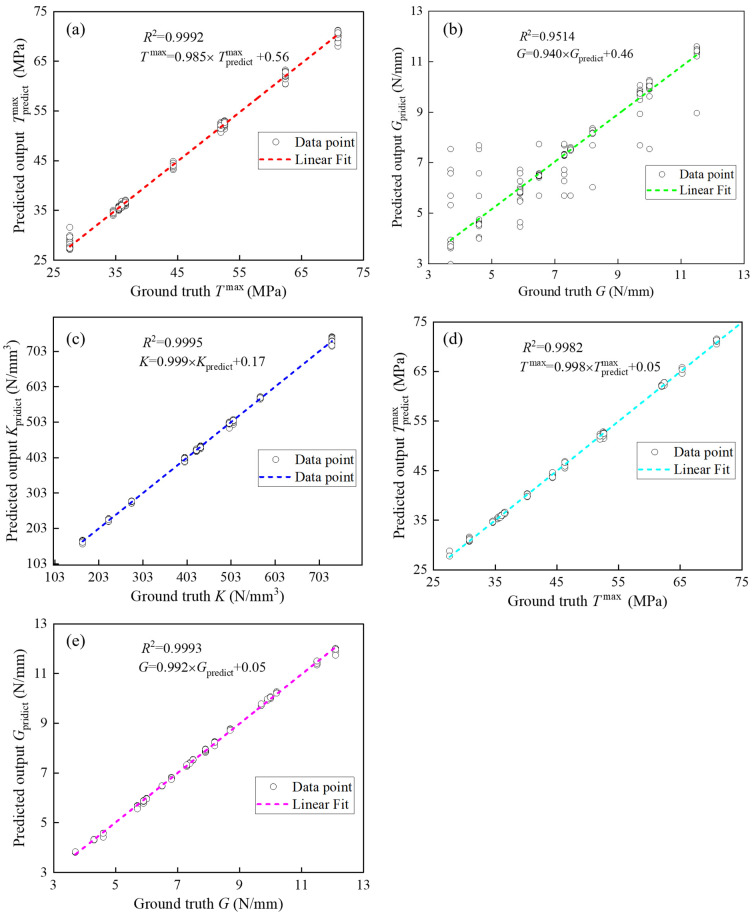
Prediction performances of bilinear and exponential law parameters: (**a**) Bil−*T*^max^; (**b**) Bil−*G*; (**c**) Bil−*K*; (**d**) exp−*T*^max^; (**e**) exp−*G*.

Figure 9 shows the prediction results of the CNN-LSTM architecture for the bilinear and exponential norm parameters on the test set. The TSL parameters are all predicted very well. Bil−*G* has the worst prediction results but is still acceptable.

Table 4 and Table 5 give the prediction ability of the three ML architectures for TSL parameters based on the performance metrics RMSE and MAPE. Comparing the prediction performance metrics of the three ML architectures for each TSL parameter, both RMSE and MAPE are minimized for the CNN-LSTM architecture, i.e., the CNN-LSTM architecture makes the best prediction. In addition, by comparing the predictive ability of the corresponding parameters of the three TSL shapes, it is found that the polynomial and exponential law parameters outperform the predictive effect of the bilinear law parameters. The above results indicate that the CNN-LSTM architecture is able to recognize TSL parameters effectively and shows a good prediction ability, especially for exponential and polynomial law parameters.

### 5.2. Model Verification

Although the CNN-LSTM architecture has been validated using a variety of statistical metrics, the performance of the architecture on experimental data has not yet been validated; therefore, the predicted results must be compared with the experimental results. When using the ML architecture built in this paper for prediction, the appropriate TSL shape and corresponding parameters can be obtained by inputting the *F*–*δ* curves obtained from the shear experiments at the bonded interface of the IGBT modules. This ML architecture can be validated by comparing the error between the FE-CZM numerical simulation results based on the given TSL shape and parameters and the experimental *F*–*δ*-curve response.

**Figure 10:**
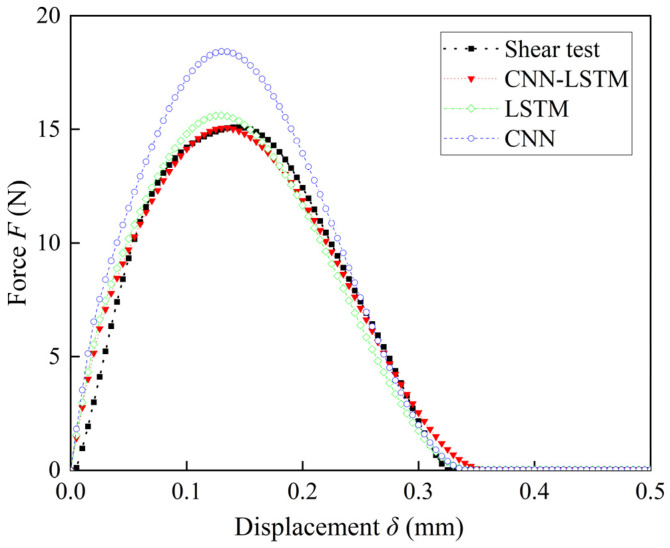
Comparison of the experimental results and the FE-CZM results obtained using TSL.

The *F*–*δ* curves are identified using the shear tests and the numerical simulation results obtained using the TSL given by the CNN, LSTM, and CNN-LSTM architectures. These curves are depicted in Figure 10. The errors between the simulation results and the experimental results are calculated using the metric RMSE. RMSE values of 3.0166, 0.6599, and 0.3815 were obtained for the CNN, LSTM, and CNN-LSTM, respectively. These findings show that the results obtained using the proposed CNN-LSTM architecture most effectively align with the experimental results and can accurately determine the TSL parameters that characterize the mechanical properties of the bonded interface of the IGBT module.

### 5.3. Effects of Cohesive Shape

**Figure 11:**
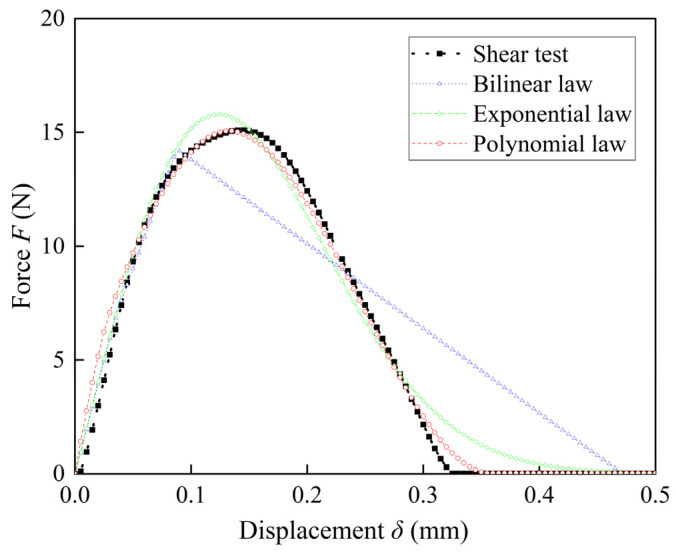
Effects of the TSL shape on the *F*–*δ* response curve.

Based on the CNN-LSTM architecture and FE-CZM models, the impacts of TSL shapes, i.e., the bilinear law, exponential law, and polynomial law, on the prediction results are discussed. Here, the CNN-LSTM architectures were first applied to obtain the TSL parameters; the TSL shape-prediction process was skipped, and the experimentally obtained *F*–*δ* curves were inputted to the CNN-LSTM architecture to directly obtain the parameters corresponding to the three TSL shapes. The results are provided in Table 6. Then, FE-CZM simulations were performed based on the parameters given in Table 6 and the corresponding TSL laws, and the *F*–*δ* response curves were obtained, as shown in Figure 11.

As shown in Figure 11, the *F*–*δ* curve exhibits the same shape as its TSL shape. This phenomenon was also found in the experimental results of a single lap joint specimen under type II loading conditions by Xu [19]. This is because the cohesive layers are considered to be subjected to nearly the same shear stress. This suggests that the effect of the TSL shape is not negligible when predicting the mechanical properties of the bonded interface for IGBT modules. The error between these results and the experimental results was calculated using the metric RMSE. RMSE values of 6.3662, 0.6896, and 0.3815 were obtained for the bilinear, exponential, and polynomial law, respectively. This result shows that the polynomial law most effectively aligns with the experimental results. The final TSL shapes and corresponding parameters suitable for characterizing the mechanical properties of the IGBT bonded interface were obtained; the polynomial law shape was selected, and the Pol−*T*^max^ is 53.763 MPa, and the Pol−*G* is 9.629 N/mm.

## 6. Conclusions

In this work, we establish an FE-CZM model for describing the mechanical behavior of the bonded interface of IGBT modules and combine it with a constructed ML architecture to accurately identify TSL shapes and corresponding parameters. The prediction performances of three architectures, CNN, LSTM, and CNN-LSTM, are compared, the influence of the TSL shape is discussed, and the effectiveness of the CNN-LSTM architecture is verified using the results of shear tests. The following conclusions are drawn from this work:

The proposed CNN-LSTM architecture accurately recognizes suitable TSL shapes, achieving an error rate of 0.186%. Compared to the CNN and LSTM architectures, this architecture achieves the lowest error rate and improves the test performance by 2−4 times.

Based on the obtained TSL shapes, the architecture can more accurately predict the corresponding TSL parameters. The TSL parameters of the three TSL shapes are predicted, and the analysis results reveal that the *R*^2^ value is greater than 0.9514, the RMSE is less than 3.6704, and the MAPE is less than 3.5044%. This strongly suggests that the CNN-LSTM architecture proposed in this work excels in recognizing TSL parameters. The CNN-LSTM architecture has the best prediction ability for each TSL parameter relative to the separate CNN and LSTM architectures.

The shear test results of the IGBT modules are used as inputs to the CNN-LSTM architecture to accurately predict the TSL shapes and parameters suitable for describing the mechanical behaviors of the bonded interfaces of IGBT modules. Comparing the *F*–*δ* curves of the FE-CZM results with the experimental results reveals an RMSE value of 0.3815, indicating the accuracy of this prediction method.

The influence of the TSL shape is also discussed. The analysis results reveal that the TSL shape significantly impacts the prediction results when the interface parameters of IGBT modules are predicted. The results yielded by using the polynomial law align best with the experimental results.

The main contribution of this paper is the proposed ML architecture, which enables suitable TSL shapes and corresponding TSL parameters to be accurately predicted and identified by experimentally obtaining the *F*–*δ* response curves of the bonding interfaces of IGBT modules. A promising and effective solution is provided for describing the mechanical behaviors of IGBT-module bonded interfaces.

The IGBT module will withstand stress and loads in different directions during the test. However, we only considered the load in the shear direction in this paper. Therefore, complex load conditions should be considered in the future.

## Figures and Tables

**Table 1:** Material properties.

Materials	Young Modulus (GPa)	Poisson’s Ratio	Yield Strength (MPa)	Tangent Modulus (MPa)
Si	112	0.22	--	--
Al	70.6	0.33	30	500

**Table 2:** Comparison of the error rates for predicting TSL shapes with the three ML architectures.

Indicators	CNN		LSTM		CNN-LSTM	
	Train	Test	Train	Test	Train	Test
Si	0	0.742	0.081	0.371	0	0.186

**Table 3:** Metrics for the performance measure of *R*^2^.

TSL Parameters	Bil−*T*^max^	Bil−*G*	Bil−*K*	Exp−*T*^max^	Exp−*G*	Pol−*T*^max^	Pol−*G*
CNN	0.9832	0.9183	0.9959	0.9864	0.9785	0.9858	0.9917
LSTM	0.99405	0.9250	0.9989	0.9971	0.9978	0.9935	0.9952
CNN-LSTM	0.9992	0.9514	0.9995	0.9982	0.9993	0.9997	0.9992

**Table 4:** Metrics for the performance measure of RMSE.

TSL Parameters	Bil−*T*^max^	Bil−*G*	Bil−*K*	Exp−*T*^max^	Exp−*G*	Pol−*T*^max^	Pol−*G*
CNN	1.7574	0.6769	4.6040	1.7160	0.3267	1.8908	0.2121
LSTM	1.0139	0.6266	5.0425	0.7832	0.1082	1.2690	0.1621
CNN-LSTM	0.3730	0.5075	3.6704	0.6468	0.0621	0.3019	0.0643

**Table 5:** Metrics for the performance measure of MAPE (%).

TSL Parameters	Bil−*T*^max^	Bil−*G*	Bil−*K*	Exp−*T*^max^	Exp−*G*	Pol−*T*^max^	Pol−*G*
CNN	1.9726	4.9376	10.1005	2.7242	2.6373	2.3980	2.8075
LSTM	1.3783	4.2750	0.9551	0.9735	1.1564	1.5895	1.2631
CNN-LSTM	0.6013	3.5044	0.7285	0.5765	0.6642	0.4141	0.5247

**Table 6:** The TSL parameters predicted by the CNN-LSTM architecture.

TSL	Cohesive Parameters		
	Stiffness *K* (N/mm^3^)	Strength *T* (MPa)	Energy *G* (N/mm)
Bilinear law	602.347	41.781	9.493
Exponential law	--	46.228	9.334
Polynomial law	--	53.763	9.629

## Data Availability

Data are contained within the article.
